# Identifying high-risk neurological phenotypes in adult-onset classic monogenic autoinflammatory diseases: when should neurologists consider testing?

**DOI:** 10.1186/s12883-024-03621-3

**Published:** 2024-04-17

**Authors:** Guilherme Diogo Silva, João Vitor Mahler, Sérgio Roberto Pereira da Silva Junior, Leonardo Oliveira Mendonça, Pedro Lucas Grangeiro de Sá Barreto Lima, Paulo Ribeiro Nóbrega, Fernando Kok, Fernando Freua

**Affiliations:** 1 Neuroimmunology Group, Division of Neurology, Hospital das Clinicas (HCFMUSP), Faculdade de Medicina, Universidade de Sao Paulo, São Paulo, Brazil; 2 Faculdade de Medicina, Universidade de Sao Paulo, São Paulo, Brazil; 3 Neurogenetics Group, Division of Neurology, Hospital das Clinicas (HCFMUSP), Faculdade de Medicina, Universidade de Sao Paulo, São Paulo, Brazil; 4 Division of Clinical Immunology and Allergy, Hospital das Clinicas (HCFMUSP), Faculdade de Medicina, Universidade de Sao Paulo, São Paulo, Brazil; 5 Faculty of Medicine, Federal University of Ceara, Fortaleza, Brazil; 6 Division of Neurology, Walter Cantídio University Hospital, Federal University of Ceará, Fortaleza, Brazil; 7 Centro Universitário Christus, Fortaleza, Brazil

**Keywords:** Autoinflammatory disease, Stroke, Meningitis, Demyelinating disease, Genetic disease

## Abstract

**Background:**

Monogenic autoinflammatory disorders result in a diverse range of neurological symptoms in adults, often leading to diagnostic delays. Despite the significance of early detection for effective treatment, the neurological manifestations of these disorders remain inadequately recognized.

**Methods:**

We conducted a systematic review searching Pubmed, Embase and Scopus for case reports and case series related to neurological manifestations in adult-onset monogenic autoinflammatory diseases. Selection criteria focused on the four most relevant adult-onset autoinflammatory diseases—deficiency of deaminase 2 (DADA2), tumor necrosis factor receptor associated periodic fever syndrome (TRAPS), cryopyrin associated periodic fever syndrome (CAPS), and familial mediterranean fever (FMF). We extracted clinical, laboratory and radiological features to propose the most common neurological phenotypes.

**Results:**

From 276 records, 28 articles were included. The median patient age was 38, with neurological symptoms appearing after a median disease duration of 5 years. Headaches, cranial nerve dysfunction, seizures, and focal neurological deficits were prevalent. Predominant phenotypes included stroke for DADA2 patients, demyelinating lesions and meningitis for FMF, and meningitis for CAPS. TRAPS had insufficient data for adequate phenotype characterization.

**Conclusion:**

Neurologists should be proactive in diagnosing monogenic autoinflammatory diseases in young adults showcasing clinical and laboratory indications of inflammation, especially when symptoms align with recurrent or chronic meningitis, small vessel disease strokes, and demyelinating lesions.

**Supplementary Information:**

The online version contains supplementary material available at 10.1186/s12883-024-03621-3.

## Introduction

Monogenic autoinflammatory disorders arise from mutations in a single gene, leading to dysregulation of the innate immune system, hyperinflammation, and various patterns of organ dysfunction, including within the central nervous system [1]. While many germinative genetic diseases typically manifest symptoms during childhood, over 10% of the germinative cases can present as an adult-onset inflammatory disease. The International Union of Immunological Societies (IUIS) classifies autoinflammatory diseases as inborn errors of immunity, and according to the latest classification, at least 56 genes have been associated with monogenic diseases, stemming from both somatic and germline mutations [2]. It is worth noting that some diseases may only manifest in adulthood, and this is particularly true for conditions such as familial Mediterranean fever (FMF), deficiency of adenosine deaminase 2 (DADA2), cryopyrin-associated periodic syndrome (CAPS), and tumor necrosis factor receptor associated periodic fever syndrome (TRAPS) [1].

Misdiagnosis and diagnostic delay pose significant challenge in autoinflammatory diseases [3], particularly in less frequent presentations such as adult-onset disease. Case series have illustrated that adults with autoinflammatory diseases can exhibit a diverse array of neurological symptoms. Such manifestations range from chronic or recurrent meningitis to cerebral infarction or hemorrhage, intracranial hypertension, and cranial nerve neuropathies [4]. Furthermore, these case series underscore the necessity of early recognition in order to initiate immunotherapy and avert irreversible neurological complications, the ultimate goal of personalized medicine [4].

Nevertheless, the neurological manifestations of monogenic autoinflammatory disorders remain an unfamiliar terrain for many neurologists, often resulting in diagnostic delays and irreversible neurological damage. Our objective in this manuscript is to provide a systematic review of the neurological manifestations of the most prevalent adult-onset autoinflammatory disorders, and to propose high-risk phenotypes for which neurologists should be vigilant and consider additional testing.

## Methods

We conducted a systematic review of published case reports and case series relating to neurological manifestations of a select group of adult-onset monogenic autoinflammatory diseases. The study was designed and conducted in adherence with the Preferred Reporting Items for Systematic Reviews and Meta-Analyses (PRISMA) guidelines to ensure comprehensive and unbiased synthesis of the available evidence [5]. The review was not pre-registered, and a protocol was not prepared in advance.

The inclusion criteria for this review were as follows: case reports or case series reporting on monogenic autoinflammatory diseases, presence of neurological manifestations in the reported patient(s), and patients aged 18 years and older. Exclusion criteria were: autoinflammatory diseases other than DADA2, TRAPS, CAPS, and FMF; and disease onset before the age of 18. Review articles were not included, although the reference lists of review articles were screened for potential additional records. There were no specific criteria for the genetic screening. Patients carrying either mono or biallelic *MEFV* mutations; germinative, somatic or mosaic mutations in *NLRP3*; and mutations in the *TNFRSF1A* (i.e. R92Q) were included.

The search strategy employed the use of synonyms for autoinflammatory diseases and neurological symptoms (see Additional file 1) in three major databases: PubMed, Embase, and Scopus. The last search run was completed in December 2022, and no restrictions were placed on language or date of publication. The search results were independently screened by two reviewers, first at the title and abstract level, then at the full-text level. In cases of disagreement, consensus was reached through discussion.

Data were extracted from the included studies using a standardized form. The data elements extracted included: author, type of autoinflammatory disease, the specific mutation, patient age and sex, age of symptom onset, family history of autoinflammatory diseases, neurological phenotype presented, neuroimaging pattern, presence of fever, presence of extra-neurological symptoms, elevation of acute phase reactants, prescribed treatment, and response to the treatment.

Summary measures were presented using mean and standard deviation for continuous variables, and percentage and total counts for categorical variables. Statistical analyses were performed using the R Studio version 2022.07.2 The four included diseases (FMF, DADA2, CAPS, and TRAPS) were compared according to their neurological phenotypes, neuroimaging findings, elevation of acute phase reactants, and the presence of fever. A *p*-value lower than 0.05 was considered statistically significant. Appropriate statistical tests, either Fisher's exact test or the Mann–Whitney U test, were employed based on the nature of the data.

## Results

**Fig. 1:**
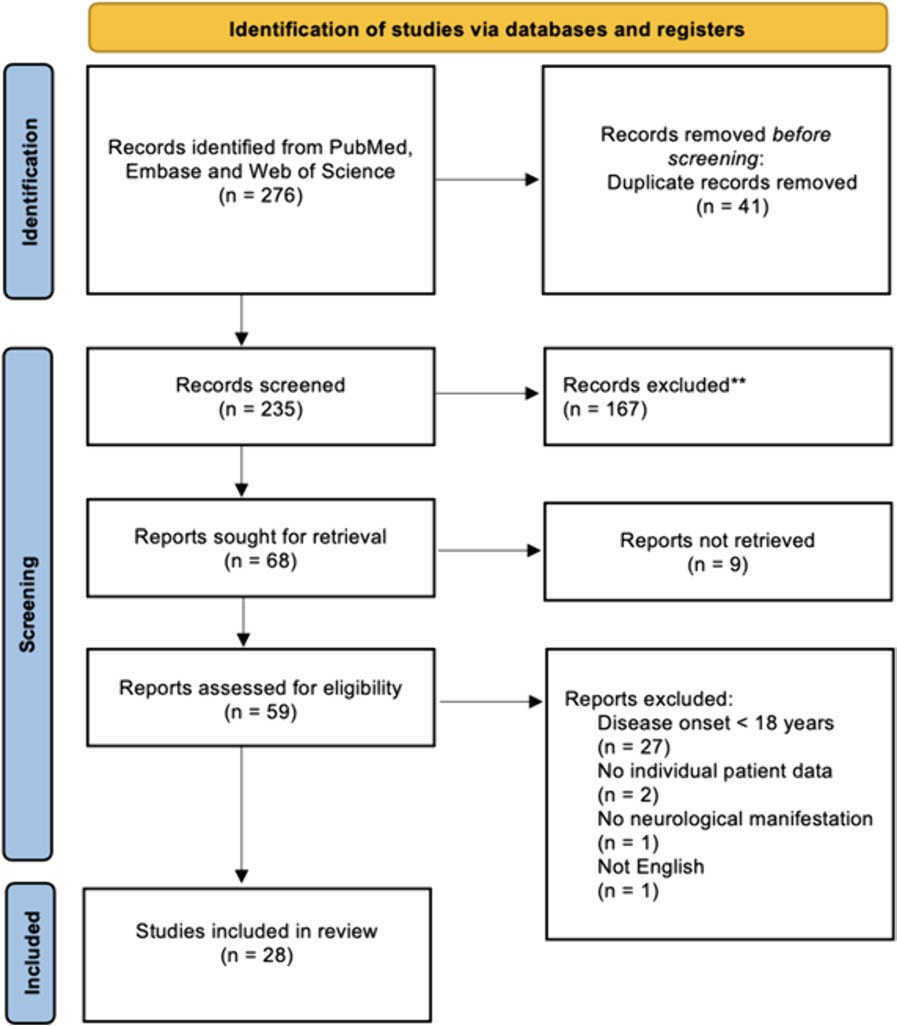
Flow diagram for article selection

We found 276 records in Pubmed, Embase and Web of Science databases, of which 41 were duplicates. After title and abstract screening, 68 manuscripts were sought for retrieval. Nine were not retrieved due to unavailability; 59 articles were read in full-text, out of which 28 were included in our review (Fig. 1) [4, 6–32].

Table 1 summarizes patient characteristics. Data from 51 patients was gathered. The median age was 38 years old and there was a male predominance (63%). Neurological manifestations occurred after a median disease duration of 5 years, although some cases presented at onset. The most common neurological symptom was headache, followed by symptoms of cranial nerve dysfunction, focal neurological deficits, and seizures. Almost all cases had a history of fever. More than 70% of the reported cases had elevated acute phase reactants such as C-reactive protein or elevated sedimentation rate. When abnormal, the three most common neuroimaging patterns were demyelinating lesions (45%), stroke (14%), and meningeal enhancement (22%).

**Table 1:** Patient demographic and clinical characteristics

Characteristic	Total (*n* = 51)	FMF (*n* = 38)	CAPS (*n* = 6)	TRAPS (*n* = 5)	DADA2 (*n* = 2)
**Demographics**					
Age at onset (years)	31.3 (11.8)	29.3 (9.9)	38.8 (16.6)	31.2 (9.2)	46 (28.3)
Gender distribution (% Female)	19 (37.2%)	16 (42.1%)	1 (16.7%)	1 (20%)	1 (50%)
**Neurological signs and symptoms**					
Headache	29 (56.9%)	19 (50%)	5 (83.3%)	5 (100%)	0
Focal deficits	14 (27.4%)	12 (31.6%)	0	0	2 (100%)
Seizures	7 (13.7%)	3 (7.9%)	3 (50%)	0	1 (50%)
Cranial nerve dysfunction	14 (27.4%)	9 (23.7%)	3 (50%)	0	1 (100%)
**Neuroimaging patterns**					
Aseptic meningitis	11 (21.6%)	8 (21%)	3 (50%)	-	0
CNS demyelination	23 (45.1%)	21 (55.3%)	2 (33.3%)	-	0
Stroke	7 (13.7%)	4 (10.5%)	1(16.7%)	-	2 (100%)
**Laboratory findings**					
Most frequently associated genes	-	MEFV	NLRP3	TNFRSF1A	ADA2 (CECR1)
Elevation of serum CRP or ESR	14 (73.7%)	9 (64.3%)	3 (100%)	-	2 (100%)

*CRP* C reactive protein, *ESR* Erythrocyte Sedimentation Rate, *MRI*: Magnetic Resonance Imaging, *DADA2* Deficiency of Deaminase 2, *FMF* Familial Mediterranean Fever, *CAPS* Cryopyrin-associated periodic syndrome, *TRAPS* Tumor necrosis factor receptor associated periodic fever syndrome

We compared the predominant phenotype of patients with DADA2, FMF and CAPS (Table 1). The most common phenotype of DADA2 was stroke (100%); the most common phenotype of FMF was demyelinating lesions (55%); and the most common phenotype of CAPS was meningitis (50%). Case reports with TRAPS had insufficient neuroimaging description to further classification.

Both ischemic and hemorrhagic stroke occurred in patients with DADA2 [26, 28]. Ischemic stroke mechanism was interpreted as cerebral small vessel disease. No patient had large vessel disease. Ischemic stroke occurred predominantly in the basal ganglia [28]. DADA2, FMF and CAPS were associated with ischemic stroke, whereas hemorrhagic stroke was only present in ADA2 mutations.

Demyelinating lesions were predominantly observed in the brain, although they were also present in the optic nerve and spinal cord [8, 10, 27, 29, 31]. We found a similar distribution of males and females among patients with demyelinating lesions (12/23, 52%). The morphology and distribution of central nervous system demyelinating lesions resembled those typically found in multiple sclerosis. These lesions met the McDonald 2017 criteria for multiple sclerosis [33] in 14 out of 21 (67%) of reported cases, all which involved FMF patients. However, we also observed less common features such as tumefactive lesions and a case of conus medullaris involvement [27]. None of the cases reported the presence of anti-MOG or anti-AQP4 autoantibodies, although there was limited information available regarding the frequency of these tests being conducted (including for the patient with conus involvement). Cerebrospinal fluid oligoclonal bands were reported in 6 out of 23 (26%) cases.

Meningitis was usually acute or subacute (duration of weeks). Recurrent aseptic meningitis was seen in patients with FMF and two cases of CAPS carrying NLRP3 mutations. In most instances, these patients displayed unremarkable MRI findings despite CSF pleocytosis, although one showed leptomeningeal involvement [32]. Interestingly, two cases (one of FMF and one of CAPS) presented with pachymeningeal involvement, particularly in the cavernous sinus [18, 21].

## Discussion

**Fig. 2:**
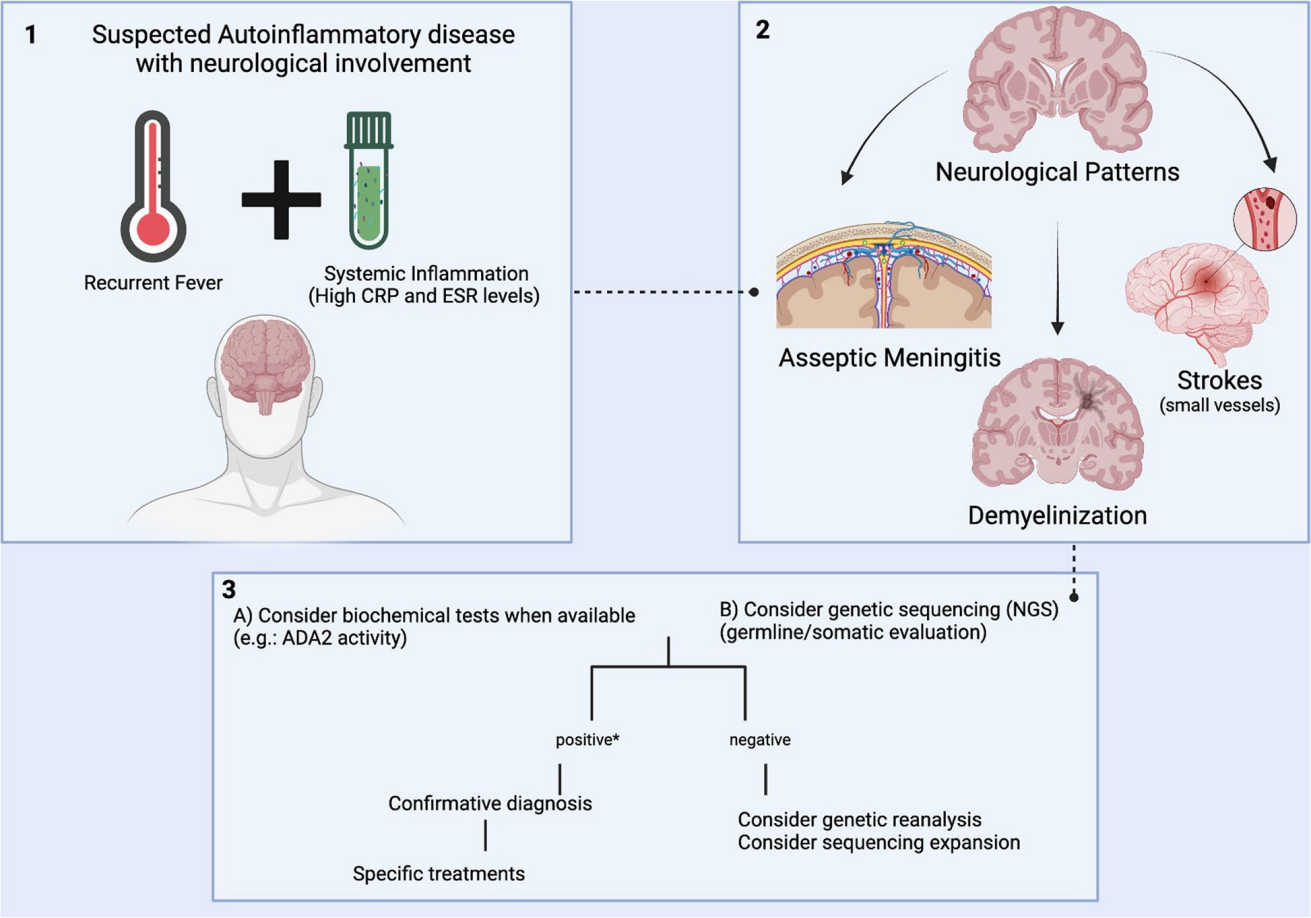
Neurological phenotype according to the autoinflammatory disease. DADA2: Deficiency of Deaminase 2; FMF: Familial Mediterranean Fever; CAPS: Cryopyrin-associated periodic syndrome; MS: Multiple Sclerosis; CRP: C Reactive Protein; ESR: Erythrocyte Sedimentation Rate

We found the neurological manifestations of these four (CAPS, TRAPS, FMF and DADA2) monogenic autoinflammatory diseases were mainly stroke, demyelinating lesion and meningitis in young adults, usually associated with evidence of systemic inflammation (e.g. fever or elevated acute phase reactants) (Fig. 2).

The first phenotype was ischemic stroke in young adults, particularly the subtype of cerebral small vessel disease, in a patient with evidence of systemic inflammation. In this setting, DADA2 was the most relevant autoinflammatory disease. The deficiency of *ADA2* leads to an increased activation of macrophages and release of cytokines that predispose to vessel wall inflammation [34]. Identifying DADA2 is paramount for neurologists because the start of anti-TNF monoclonal antibodies is associated with reduction of the stroke recurrence, control of systemic inflammation and prevention of additional relapses. Furthermore, the correct diagnosis can directly impact patient outcomes because the use of aspirin, a standard treatment for ischemic strokes of undetermined subtype, has raised controversy in the context of DADA2 patients due to reported cases of hemorrhagic strokes associated with antithrombotic therapy [35].

The second phenotype was multiple sclerosis-like demyelinating lesions, in patients with evidence of systemic inflammation. There is uncertainty whether the demyelination corresponds to an autoimmune comorbidity or a manifestation of FMF. This subject has sparked debate, driven by important changes in the diagnostic criteria of FMF over the last decade—transitioning from exclusive reliance on clinical manifestations to an objective genetic diagnosis. Currently, the most accepted diagnostic criteria do not include any neurological manifestations and the final diagnosis is typically established after unveiling biallelic mutations in the *MEFV* gene. Notably, this evolution in diagnostic criteria may introduce certain limitations into our study, as historical reports may not align with the contemporary classification criteria for FMF. Although heterozygosity for the MEFV gene is not associated with multiple sclerosis, its diagnosis seems to be more frequent in patients with homozygous or compound heterozygous FMF [25]. Moreover, multiple sclerosis is more likely to lack oligoclonal bands when associated with FMF, and treatments for FMF such as colchicine and anti-IL 1 monoclonal antibodies prevent new demyelinating lesions in case reports [25]. The absence of a female preponderance and the low frequency of cerebrospinal oligoclonal bands observed were atypical for MS in patients with FMF and demyelinating lesions in our study. Moreover, a third of participants with demyelinating lesions did not meet the 2017 McDonald diagnostic criteria for MS [33], which may indicate the presence of white matter lesions with morphology and distribution that are atypical for MS and the lower frequency of oligoclonal bands. The limited information available regarding anti-MOG or anti-AQP4 antibody tests raises concerns for cases that do not meet the McDonald 2017 diagnostic criteria, especially considering that oligoclonal band-negative cases are more common in MOG-associated antibody disease and neuromyelitis optica spectrum disorders. Future studies should address this knowledge gap.

The third phenotype was recurrent aseptic meningitis, both in patients with FMF and CAPS. CSF inflammation in autoinflammatory diseases may result from increased cytokine production, mainly IL-1β and IL-6, by microglia and astrocytes due to inflammasome dysfunction [36]. In a large series of CAPS (*n* = 136), 26% of patients exhibited aseptic meningitis [37]. Leptomeningitis emerged as the predominant phenotype, accounting for all cases except for two patients with pachymeningitis, reminiscent of the Tolosa-Hunt syndrome. Additionally, in a case series of neurological manifestations of autoinflammatory diseases in Chinese adult patients (*n* = 31), pachymeningitis in the tentorium cerebellum was identified in a patient with an NLPR3 mutation [4]. Further research should explore the potential significance of genetic testing in individuals diagnosed with idiopathic pachymeningitis. Chronic meningitis is traditionally linked to mycobacterial and fungal infections; however, there is an increasing awareness of non-infectious causes [38–40]. Recognition of autoinflammatory diseases as causes of recurrent or chronic meningitis is important because colchicine and anti-IL 1 monoclonal antibodies may prevent new flares of meningitis.

Our study has some limitations. First, some articles did not present detailed genetic data and, hence, we could not perform the standardized classification by the American College of Medical Genetics (ACMG) to classify the genomic variant according to its pathogenicity. Hence, it is possible that non-pathogenic variants may have been included in this review. Most identified cases presented FMF and, hence, other autoinflammatory neurological disorders were underrepresented. This finding may represent the epidemiology of autoinflammatory disorders, considering the significantly higher worldwide prevalence of FMF (1 to 5 per 10,000) when compared to CAPS (1 per 1,000,000) or TRAPS (more than 1,000 cases) [41].

Moreover, there is a possibility of publication bias, as more severe neurological manifestations are more likely to be published than mild manifestations of diseases. Moreover, our study design, which included only case reports wherein neurological symptoms were reported, introduced a selection bias, and may decrease the accuracy of the rate of each neurological presentation. For this reason, future observational prospective multicentric studies should perform standardized neurological evaluation of patients with autoinflammatory diseases to improve that characterization of the neurological phenotypes of these diseases.

## Conclusion

Neurologists should actively seek the diagnosis of monogenic autoinflammatory diseases in young adults displaying clinical and laboratory indicators of inflammation, especially in cases involving small vessel strokes, demyelinating lesions, and recurrent or chronic meningitis. Testing should be pursued selectively when specific red flags are evident, with recurrent fever being a prominent feature present in the majority of cases.

### Supplementary Information

**Supplementary Material 1.**

## Data Availability

All data available is contained in the manuscript and additional information.
