# Supplementary material for: Identifying high-risk neurological phenotypes in adult-onset classic monogenic autoinflammatory diseases: when should neurologists consider testing?
Source: BMC Neurol. 2024 Apr 17;24:130. doi: 10.1186/s12883-024-03621-3 (PMC11022464; doi:10.1186/s12883-024-03621-3)
Supplement: Supplementary file 1 — Supplementary Material 1. [file 12883_2024_3621_MOESM1_ESM.docx]

**Additional File 1**

**File name:** Additional File 1

**File format:** .docx

**Title of Data:** Search strategy

**Description of data:** Search strategies used in databases for systematic review.

("autoinflammatory disease" OR "autoinflammatory disorder" OR "Cryopyrin associated periodic syndrome" OR "deficiency of adenosine deaminase 2" OR "Familial Mediterranean Fever" OR "TNF receptor-associated periodic syndrome") AND ("Neurological manifestation" OR “Neurological presentation" OR "Brain" OR "Spinal Cord" OR "Meninges" OR "Cranial nerve" OR "Peripheral nerve" OR "Neuropathy"OR "Stroke" OR "Demyelinating disease" OR "Meningitis" OR "Encephalitis" OR "Myelitis" OR "Neuritis" OR "Headache" OR "Seizure" OR "Vertigo" OR "Cognitive impairment" OR "Encephalopathy" OR "Movement disorder") AND ("Case Reports" OR "Case series")

Database: Pubmed

Last run: December 23th, 2022

('autoinflammatory disease' OR 'autoinflammatory disorder' OR 'Cryopyrin associated periodic syndrome' OR 'deficiency of adenosine deaminase 2' OR 'Familial Mediterranean Fever' OR 'TNF receptor-associated periodic syndrome' ) AND (Brain OR 'Spinal cord' OR Meninges OR Neuropathy OR Stroke OR 'Demyelinating disease' OR Seizure OR Vertigo OR 'Movement disorder' OR 'Cognitive impairment') AND ('Case Reports' OR 'Case series' )

Database: Embase

Last run: December 23th, 2022

('autoinflammatory disease' OR 'autoinflammatory disorder' OR 'Cryopyrin associated periodic syndrome' OR 'deficiency of adenosine deaminase 2' OR 'Familial Mediterranean Fever' OR 'TNF receptor-associated periodic syndrome' ) AND (Brain OR 'Spinal cord' OR Meninges OR Neuropathy OR Stroke OR 'Demyelinating disease' OR Seizure OR Vertigo OR 'Movement disorder' OR 'Cognitive impairment') AND ('Case Reports' OR 'Case series' )

Database: Web of Science

Last run: December 23th, 2022
